# Identification of a novel betaherpesvirus in *Mus musculus*

**DOI:** 10.1186/1743-422X-6-225

**Published:** 2009-12-21

**Authors:** Alla Teterina, Dania Richter, Franz-Rainer Matuschka, Bernhard Ehlers, Sebastian Voigt

**Affiliations:** 1 Research group "Molecular Genetics and Epidemiology of Herpesviruses", Robert Koch Institute, Berlin, Germany; 2 Institute for Pathology, Charité Universitätsmedizin Berlin, Berlin, Germany; 3 Division of Viral Infections, Robert Koch Institute, Berlin, Germany

## Abstract

Rodent betaherpesviruses vary considerably in genomic content, and these variations can result in a distinct pathogenicity. Therefore, the identification of unknown betaherpesviruses in house mice (*Mus musculus*), the most important rodent host species in basic research, is of importance. During a search for novel herpesviruses in house mice using herpesvirus consensus PCR and attempts to isolate viruses in tissue culture, we identified a previously unknown betaherpesvirus. The primary PCR search in mouse organs revealed the presence of known strains of murine cytomegalovirus (*Murid herpesvirus 1*) and of *Mus musculus *rhadinovirus 1 only. However, the novel virus was detected after incubation of organ pieces in fibroblast tissue culture and subsequent PCR analysis of the supernatants. Long-distance PCR amplification including the DNA polymerase and glycoprotein B genes revealed a 3.4 kb sequence that was similar to sequences of rodent cytomegaloviruses. Pairwise sequence comparisons and phylogenetic analyses showed that this newly identified murine virus is most similar to the English isolate of rat cytomegalovirus, thereby raising the possibility that two distinct CMV lineages have evolved in both *Mus musculus *and *Rattus norvegicus*.

## Findings

Cytomegalovirus (CMV), a member of the *Betaherpesvirinae*, can cause severe infections in immunocompromised hosts. Because of their inherent species-specificity, CMVs are used in rodent infection models to study human CMV (HCMV) pathogenesis. These models include, e.g., the house mouse (*Mus musculus*) and the rat (*Rattus norvegicus*) with their host-specific viruses (mouse and rat CMV; MCMV and RCMV, respectively). CMVs have evolved with their hosts for a long period of time, and this coexistence has shaped their genetic content. In addition, frequent serial passaging of the commonly used MCMV laboratory strains Smith and K181 has resulted in even further variations of viral genes and the emergence of new genotypes [1-4]. This genetic variation, including the loss of numerous genes, has been also reported for clinical isolates of HCMV [5,6].

**Figure 1:**
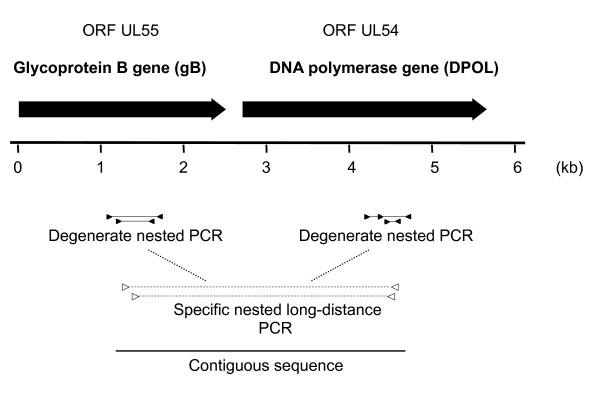
**Amplification strategy**. At the top of the figure above the ruler, the ORFs of UL55 and UL54 are represented by black arrows. Below the ruler, the PCRs are depicted. The degenerate and specific primers are represented by black and open triangles, respectively. Amplified sequences are shown as black lines.

To determine the impact that the presence of modified or completely different genes on CMV biology might imply, we sought to detect novel betaherpesviruses. To do so, we employed consensus PCR with degenerate primers that had previously proven useful in identifying a hitherto unknown gammaherpesvirus, the first reported rhadinovirus in *Mus musculus *[7]. Spleens, lungs, inguinal lymph nodes and salivary glands were obtained from two or three individuals of four strains of house mice. Each organ sample was divided into two pieces, one for DNA extraction followed by an initial PCR analysis with degenerate consensus primers and one for virus isolation in fibroblast tissue culture. The organ samples were minced and co-cultivated on monolayers of murine 3T3 and 10.1 cells, as well as on L929 and BHK21 cells. About two weeks later, supernatants were taken, DNA was extracted, and another PCR analysis was performed using the same primers as in the initial analysis. DNA of organs and tissue supernatants was extracted using the QiAamp tissue kit according to the manufacturer's instructions (Qiagen, Hilden, Germany). Panherpes consensus-PCR for amplification of a 160 bp - 181 bp fragment (without primer-binding sites) of the DPOL gene [8] was carried out with five degenerate/deoxyinosine-substituted (deg/dI) nested-primers as described previously [9] (Figure 1).

**Figure 2:**
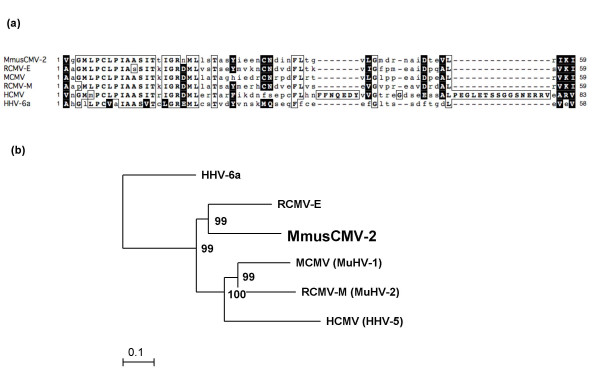
**The novel cytomegalovirus of *Mus musculus*: Multiple sequence alignment and phylogenetic analysis**. (a) The MmusCMV-2 DPOL sequence amplified by panherpes consensus PCR (178 bp) was translated into a 59 aa sequence, and a multiple sequence alignment with the corresponding sequences of MCMV, RCMV-M, RCMV-E, HCMV and HHV-6b was generated using the ClustalW module of MacVector™ 10.6. Identical and similar amino acids are boxed, the last in inverse type. Mismatches are given in lower type. (b) A phylogenetic tree was constructed using the nucleic acid sequences encoded by the gB-DPOL segments of MmusCMV-2 and those of known human, mouse and rat cytomegaloviruses (GenBank accession numbers in the *Findings *section). Abbreviations of common names are used, and those of species names according to the ICTV (International Committee on the Taxonomy of Viruses) are given in parentheses. A multiple alignment of 3.4 kb was analysed with the neighbour-joining method. A rooted phylogram is shown, with HHV-6a as outgroup. The branch length is proportional to evolutionary distance (scale bar). Results of bootstrap analysis (1000 replicates) are indicated at the nodes of the tree. The novel MmusCMV-2 is highlighted in bold type.

Direct consensus PCR analysis of 44 organ samples collected from eleven mice of four house mouse strains revealed the presence of several variants of MCMV in two mouse strains and of the previously described *Mus musculus *rhadinovirus 1 (MmusRHV-1 [7]) in one strain. Subsequent PCR analysis of tissue culture supernatants of MCMV-positive organs confirmed known MCMV in all cases. The tissue culture supernatants of MmusRHV-1-positive organs, however, did not contain the expected sequences, indicating that MmusRHV-1 failed to grow in cell culture. Instead, we detected a DPOL sequence of an unknown betaherpesvirus in the supernatant of L929 cells that had been co-cultivated with the MmusRHV-1-positive lung (specimen #5479) of one mouse (Figure 2a).

To amplify glycoprotein B (gB) gene sequences of that virus, the deg/dI nested-primer set CMV-gB-1 was used (Figure 1a). With this set, gB gene sequences of several members of the *Betaherpesvirinae *subfamily had previously been amplified, resulting in a second-round amplification product of approximately 225 bp (without primer-binding sites) [7]. PCR products were obtained from the L929 tissue culture supernatants, and sequencing revealed a gB sequence of an unknown betaherpesvirus.

To prove that the gB and the DPOL sequence originated from the same virus, we connected them with Long-distance (LD) PCR using the TaKaRa-Ex PCR system (Takara Bio inc., Otsu, Japan) according to the manufacturer's instructions. An amplification product of approximately 3,3 kb size was obtained and sequenced by primer walking. A contiguous sequence of 3431 bp was obtained (in combination with the initial gB and DPOL sequences), spanning 1101 bp of the 3'-part of the gB gene and 2313 bp of the 5'-part of the DPOL gene (Figure 1a). Since a cytomegalovirus of the house mouse is already known (MCMV = MuHV-1), the virus from which the novel sequence originated was tentatively named *Mus musculus *cytomegalovirus 2 (MmusCMV-2).

Rowe and Capps [10] discovered a mouse virus which causes thymic necrosis in newborn mice, however, it could not be propagated in cell culture. This virus was named mouse thymic virus (MTV) and classified as *Murid herpesvirus 3 *(MuHV-3). No sequences of MuHV3 are available in public databases. Seroepidemiological studies have shown that MuHV-3 (like MCMV) is ubiquitously present in free-living European house mice [11]. To rule out that MmusCMV-2 might in fact be MuHV-3, we compared it with a recently amplified partial DPOL sequence of MuHV-3 (R. S. Livingston, University of Missouri-Columbia, USA; personal communication). This 450 bp betaherpesvirus-like sequence of MuHV-3 was only distantly related to the corresponding DPOL sequence of MmusCMV-2 (and those of MCMV, RCMV-E and RCMV-M) (data not shown). Therefore, we concluded that MmusCMV-2 and MuHV-3 are different viruses.

From the DPOL sequence, we deduced primers specific for MmusCMV-2 (MmusCMV2-fwd: 5' CGGCATGCTCCCTTGTCTTC-3'; MmusCMV2-rev: 5'TTGATGCGAAGGACTTCGGT-3'). The expected amplification product had a size of 168 bp. With an annealing temperature of 60°C, all 44 organ samples were retested, using the MmusCMV-2-positive supernatant as a control. As expected, the cell culture supernatant tested positive for MmusCMV-2 as did lung, spleen, salivary gland and lymph node of that mouse from which the positive supernatant originated. In contrast, the organs of all other mice were negative for MmusCMV-2. Additionally, lung specimens of the MmusCMV2-positive house mouse strain and of wild house mice, caught in Lower Saxony, Germany, were tested with the MmusCMV2-specific PCR. Two mice each revealed amplification products of the expected size. These were verified to originate from MmusCMV2 by sequencing (data not shown).

To determine the evolutionary position of MmusCMV-2, the 3421 bp sequence [GenBank: GU017485] was pairwise compared with the corresponding DPOL and gB sequences of MCMV (*Murid herpesvirus 1*; [GenBank: NC_004065][3,12]), RCMV Maastricht isolate (*Murid herpesvirus 2*; [GenBank: AF232689][13,14]), RCMV English isolate (RCMV-E; [GenBank: GU018179]) and HCMV (*Human herpesvirus 5*; [GenBank: AY446894]). For MmusCMV2, pairwise identity percentages were 57.6%, 56.2%, 65.9% and 46.9%, respectively (Table 1). Therefore, we conclude that RCMV-E might be the closest relative of MmusCMV-2.

**Table 1:** Percentages of nucleic acid identity

	MmusCMV-2	RCMV-E	MCMV	RCMV-M	HCMV	HHV-6a
**MmusCMV-2**	100.0	65.9	57.6	56.2	46.9	50.1
**RCMV-E**	65.9	100.0	59.9	57.5	46.3	50.7
**MCMV**	57.6	59.9	100.0	66.8	54.2	47.4
**RCMV-M**	56.2	57.5	66.8	100.0	55.0	44.9
**HCMV**	46.9	46.3	54.2	55.0	100.0	41.6
**HHV-6a**	50.1	50.7	47.4	44.9	41.6	100.0

Multiple sequence alignments were then generated with the complete 3.4 kb nucleic acid (NA)-sequence, and a phylogenetic tree was constructed using the neighbour-joining module of the MacVector™ software (version 10.6). In the tree, MmusCMV-2 branched with RCMV-E, and MCMV (MuHV-1) with RCMV-M (MuHV-2) (Figure 2b). In parsimonial analysis with concatenated amino acid (aa) sequences (367 aa of gB plus 771 aa of DPOL) using the PARS module of the PHYLIP program package on the Trex server http://www.trex.uqam.ca, MmusCMV-2 branched with RCMV-E and RCMV-M (MuHV-2) (data not shown).

Our findings revealed that *Mus musculus *- like *Rattus norvegicus *- harbours two distinct CMV (MCMV and MmusCMV-2). The results of the pairwise sequence comparisons and the NA-based phylogenetic analysis showed that this newly identified murine virus MmusCMV-2 is most similar to RCMV-E. This raises the possibility that two distinct CMV lineages in both *Mus musculus *and *Rattus norvegicus *have evolved. At this point, however, we tentatively state that MmusCMV-2 represents a distinct CMV, as has been reported for RCMV-E and RCMV-M [15,16]. As soon as the virus has been cultured to sufficiently high titres, the complete MmusCMV-2 sequence derived from purified virus DNA may confirm its distinctiveness.

Further analysis of the molecular biology of MmusCMV-2 and the study of its behaviour *in vivo *might reveal significant differences to MCMV and the two rat CMVs and broaden our understanding of cytomegalovirus biology.

## Abbreviations

BHK: baby hamster kidney; CMV: cytomegalovirus; DPOL: DNA polymerase; gB: glycoprotein B; HCMV: human cytomegalovirus; MCMV: murine cytomegalovirus; MuHV-1: Murid herpesvirus 1; MuHV-2: Murid herpesvirus 2; MuHV-3: Murid herpesvirus 3; MmusCMV-2: *Mus musculus *cytomegalovirus 2; MmusRHV-1: *Mus musculus *rhadinovirus 1; RCMV: rat cytomegalovirus; RCMV-E: English isolate; RCMV-M: Maastricht isolate.

## Competing interests

The authors declare that they have no competing interests.

## Authors' contributions

BE, AT, DR, FRM and SV designed research; AT performed research; BE, AT and SV analysed data; and BE and SV wrote the manuscript. All authors read and approved the final manuscript.
